# Risk of malignant skin neoplasms in a cohort of workers occupationally exposed to ionizing radiation at low dose rates

**DOI:** 10.1371/journal.pone.0205060

**Published:** 2018-10-05

**Authors:** Tamara V. Azizova, Maria V. Bannikova, Evgeniya S. Grigoryeva, Valentina L. Rybkina

**Affiliations:** Clinical Department, Southern Urals Biophysics Institute (SUBI), Ozyorsk, Chelyabinsk region, Russia; Kagoshima University Graduate School of Medical and Dental Sciences, JAPAN

## Abstract

Recently an increasing trend in skin cancer rates has been observed in various populations including those exposed to different radiation types. Risk and dose-response following prolonged radiation exposure remain unclear. The present study was aimed to assess skin melanoma (SM) and non-melanoma skin cancer (NMSC) incidence risks in a cohort of workers occupationally exposed to ionizing radiation at low dose rates over prolonged periods. The study cohort included workers of a Russian nuclear production facility, Mayak Production Association (PA), who were first employed in 1948–1982 and followed up till the end of 2013 (the total of 22,377 individuals with 25% of females). Using AMFIT module of EPICURE software, relative risk and excess relative risk per unit dose (RR and ERR/Sv) were calculated. 60 SM and 294 NMSC cases were registered in members of the study cohort. SM and NMSC incidence was dependent on sex, attained age, age at first employment at the enterprise, type of facility, education level and was not dependent on calendar period of first employment, calendar period of diagnosis, duration of employment, smoking and alcohol consumption statuses. The risk of NMSC incidence was found to be significantly increased in workers occupationally exposed to ionizing radiation at cumulative doses above 2.0 Sv (RR = 2.52; 95% CI: 1.60, 3.97) compared to a reference dose category (0–0.05 Sv). NMSC incidence was found to be significantly associated with cumulative external gamma-dose with ERR/Sv of 0.49 (95% CI: 0.22, 0.90) without an adjustment for neutron dose and 0.51 (95% CI: 0.22, 0.93) while adjusted for neutron dose. Results of the analysis did not reveal a significant association of SM incidence with cumulative dose from external gamma-rays with ERR/Sv of 0.22 (95% CI: -0.29, 1.46) not including a neutron dose adjustment and of 0.15 (95% CI: -0.41, 1.31) while adjusted for dose from neutron exposure.

## Introduction

Malignant skin neoplasms (MSN) including skin melanomas (SM) and non-melanoma skin cancers (NMSC) are the most common malignancies tending to increase during the recent decades consistently [1–3], The main factors contributing to MSN are sex, age, genetic susceptibility, skin phenotype, UV-exposure, etc [3–7]. MSN risks were reported for various cohorts of individuals exposed to different types of radiation [3,7–13]. A review by the UK Independent Advisory Group on Ionising Radiation^3^ gives a detailed systematic and critical review of skin cancer studies considering a range of exposure scenarios. The authors conclude that dose-effect studies are limited due to the lack of dosimetry data. Risk estimates and dose-response model type remain unclear for prolonged exposure at low dose rates. Thus, this study as aimed to assess SM and NMSC incidence risks in a cohort of Mayak Production Association (PA) workers occupationally exposed to radiation over prolonged periods at low dose rates. Earlier, studies of this cohort demonstrated increased radiogenic incidence and mortality risks for leukemia and solid cancers, lung, liver and bone cancers [14–20].

## Materials and methods

The present record-based epidemiological study did not require any contact with cohort members. The study was reviewed and approved by Institutional Review Board (IRB) of the Southern Urals Biophysics Institute. SUBI IRB confirmed that no signed consents were needed from members of the study cohort. The study was performed in accordance with the Declaration of Helsinki.

### Study cohort

This is a retrospective cohort study. The study cohort included all workers of the Mayak PA–the first large-scale atomic industry production facility located in the Southern Urals close to Ozyorsk city, first employed at one of the main plants (reactors, radiochemical or plutonium-production plants) between 01 January 1948 and 31 December 1982 regardless of sex, age, ethnicity, occupation, duration of employment or other characteristics, in total, 22 377 individuals (with 25.4% of females).

The cohort follow-up started with a date of first employment at one of the main plants and continued till the first of the following dates: MSN diagnosis date, date of death, 31 December 2013 for alive workers residing in Ozyorsk (residents), date of ‘the last medical report’ for workers-residents with unknown vital status and for workers who had moved out of Ozyorsk (migrants).

Table 1 presents detailed characteristics of the study cohort. The mean age at start of Mayak PA employment was 24.11±7.13 years (±standard deviation) in males and 27.32±7.97 years in females. Duration of employment varied from 1 month to 60 years and was averaged as 18.04±14.28 years with only 4.7% of Mayak PA workers having been employed less than 1 year.

**Table 1:** Characteristics of the study Mayak PA worker cohort.

*Workers distribution by age at first employment*						
Age at first employment, years	Males		Females		Both sexes	
	Number	%	Number	%	Number	%
<20	5 399	32.35	771	13.55	6 170	27.57
[20–30)	8 470	50.76	3 108	54.63	11 578	51.74
[30+	2 819	16.89	1 810	31.82	4 629	20.69
Total	16 688	100.00	5 689	100.00	22 377	100.00
*Workers distribution by period of first employment*						
Period of first employment, years	Males		Females		Both sexes	
	Number	%	Number	%	Number	%
1948–1958	8 718	52.24	3 579	62.92	12 297	54.95
1959–1982	7 970	47.76	2 110	37.08	10 080	45.05
Total	16 688	100.00	5 689	100.00	22 377	100.00
*Workers distribution by facility type*						
Facility type	Males		Females		Both sexes	
	Number	%	Number	%	Number	%
Reactors	4 194	25.13	1 170	20.57	5 364	23.97
Radiochemical plant	6 857	41.09	2 360	41.48	9 217	41.19
Plutonium production plant	5 637	33.78	2 159	37.95	7 796	34.84
Total	16 688	100.00	5 689	100.00	22 377	100.00
*Workers distribution by vital status as of 31 December 2013*						
Vital status	Males		Females		Both sexes	
	Number	%	Number	%	Number	%
Deceased	10 273	61.56	2 929	51.49	13 202	59.00
Alive	5 612	33.63	2 522	44.33	8 134	36.35
Unknown	803	4.81	238	4.18	1 041	4.65
Total	16 688	100.00	5 689	100.00	22 377	100.00
*Distribution of deceased workers by attained age at death*						
Age as of exit of the study, years	Males		Females		Both sexes	
	Number	%	Number	%	Number	%
<50	1 795	17.47	172	5.87	1 967	14.90
[50–60)	2 333	22.71	353	12.05	2 686	20.35
[60–70)	3 059	29.78	634	21.65	3 693	27.97
[70+	3 086	30.04	1 770	60.43	4 856	36.78
Total	10 273	100.00	2 929	100.00	13 202	100.00
*Distribution of workers known to be alive as of 31 December 2013 by attained age*						
Age as of 31 December 2013, years	Males		Females		Both sexes	
	Number	%	Number	%	Number	%
<50	22	0.39	0	0.00	22	0.27
[50–60)	1 387	24.71	187	7.41	1 574	19.35
[60–70)	1 336	23.81	386	15.31	1 722	21.17
[70+	2 867	51.09	1 949	77.28	4 816	59.21
Total	5 612	100.00	2 522	100.00	8 134	100.00
*Workers distribution by alcohol consumption*						
Alcohol consumption	Males		Females		Both sexes	
	Number	%	Number	%	Number	%
Non-drinker	944	5.66	3 050	53.61	3 994	17.85
Moderate drinker	9 683	58.02	1 831	32.18	11 514	51.45
Heavy drinker	3 262	19.55	174	3.06	3 436	15.36
Unknown	2 799	16.77	634	11.14	3 433	15.34
Total	16 688	100.00	5 689	100.00	22 377	100.00
*Workers distribution by smoking*						
Smoking	Males		Females		Both sexes	
	Number	%	Number	%	Number	%
Never smoker	3 562	21.34	4 991	87.73	8 553	38.22
Ever smoker	12 103	72.53	280	4.92	12 383	55.34
Unknown	1 023	6.13	418	7.35	1 441	6.44
Total	16 688	100.00	5 689	100.00	22 377	100.00
*Workers distribution by education*						
Education	Males		Females		Both sexes	
	Number	%	Number	%	Number	%
Non-higher education	11 310	67.77	4 080	71.72	15 390	68.78
Higher education	3 059	18.33	750	13.18	3 809	17.02
Unknown	2 319	13.90	859	15.10	3 178	14.20
Total	16 688	100.00	5 689	100.00	22 377	100.00
*Workers distribution by race*						
Race	Males		Females		Both sexes	
	Number	%	Number	%	Number	%
Caucasian	13 556	81.23	4 604	80.93	18 160	81.15
Mixed	533	3.19	153	2.69	686	3.07
Mongoloid	0	0.00	0	0.00	0	0.00
Unknown	2 599	15.57	932	16.38	3 531	15.78
Total	16 688	100.00	5 689	100.00	22 377	100.00
*Workers distribution by duration of employment at one of the main*						
Duration of employment, years	Males		Females		Both sexes	
	Number	%	Number	%	Number	%
< 1	839	5.03	217	3.81	1 056	4.72
[1–10)	6 149	36.85	2 012	35.37	8 161	36.47
[10+	9 700	58.12	3 460	60.82	13 160	58.81
Total	16 688	100.00	5 689	100.00	22 377	100.00

By the end of the follow-up vital status was known for 95% of the study cohort members with 53.5% deceased and 46.5% alive. The mean age at death was 61.52±13.63 years in males and 70.4±12.44 in females with the age of alive workers averaged at 68.50±10.40 and 76.59±9.75 years, respectively.

In the present study MSN risks were assessed, namely SM (ICD-9 codes 172.0–172.9) and NMSC including basal cell skin cancer, squamous cell carcinoma and others (ICD-9 codes 173.0–173.9) [21]. As reported earlier 22, all Mayak PA workers were subjected to a preliminary medical health examination (prior to employment) and to annual medical health checks which included a dermatologist examination over the whole period of employment at the facility. Using medical and dosimetry database ‘Clinic’ [22], 354 primary MSNs were identified in members of the Mayak worker cohort (224 (63.3%) registered in males and 130 (36.7%) registered in females). It should be noted, that 100% of identified cases were verified based on results of histological examination.

### Dosimetry

**Fig 1:**
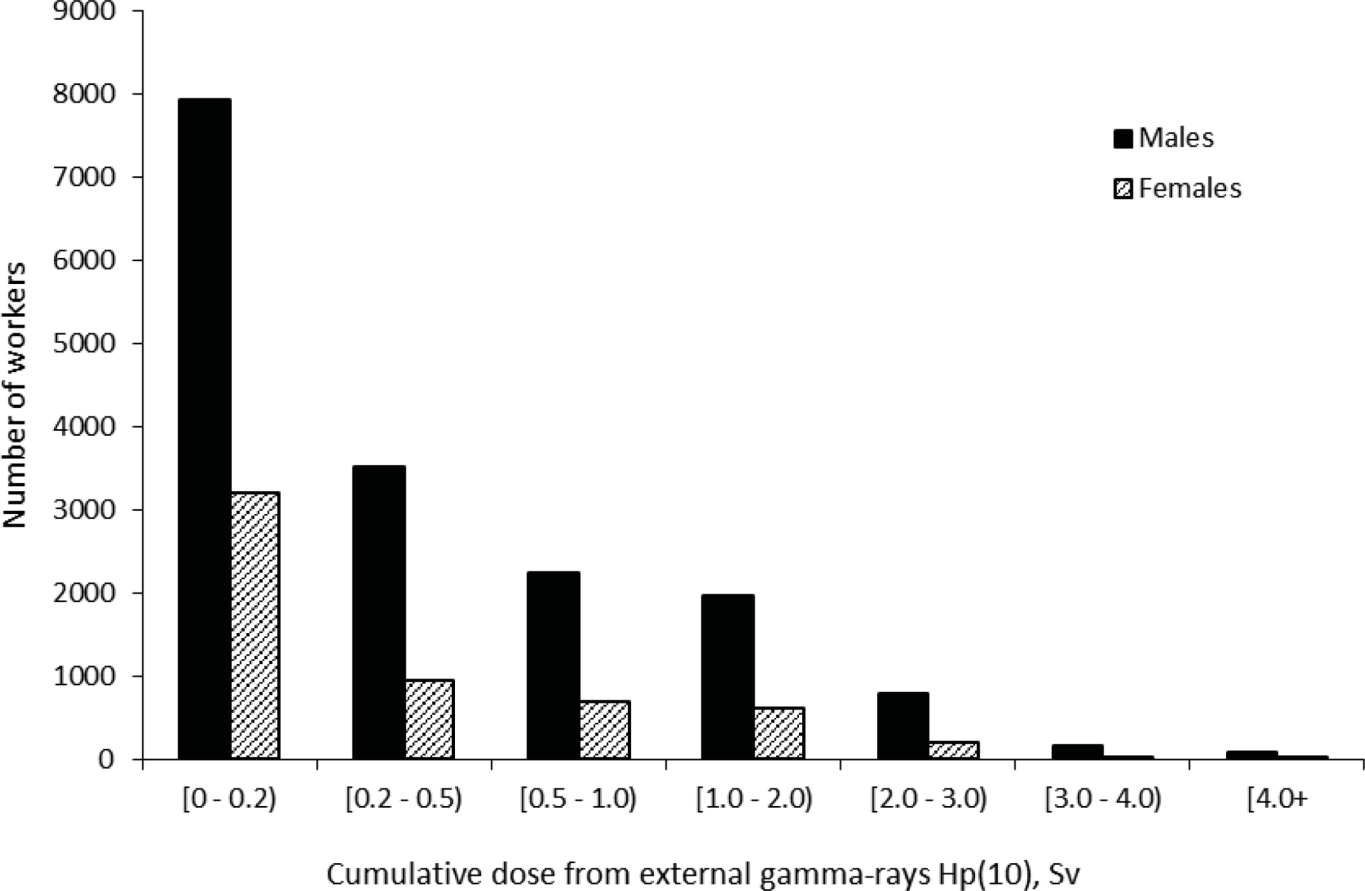
Distributions of workers from the study cohort depending on cumulative dose from external gamma-rays.

**Fig 2:**
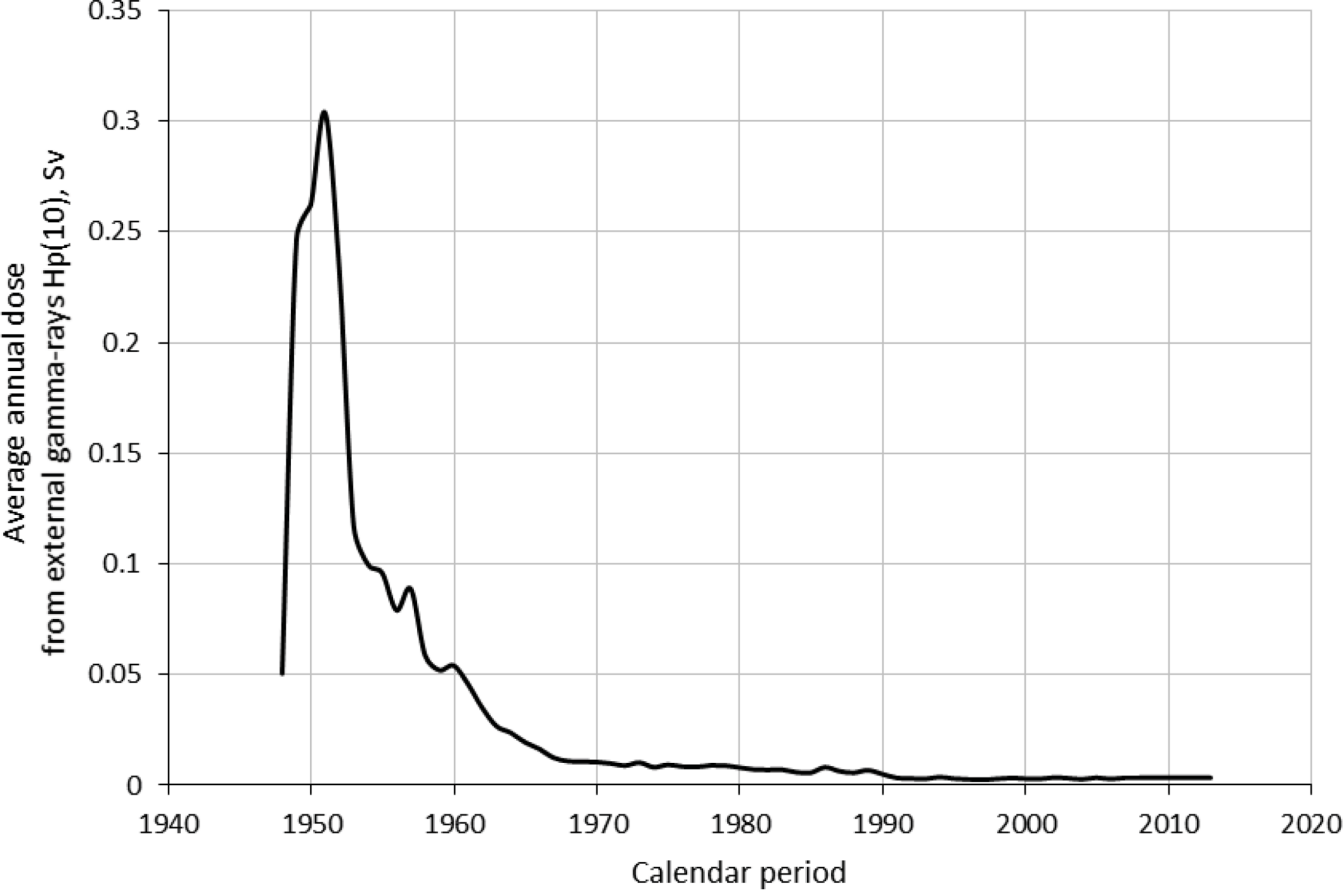
Average annual dose from external gamma-rays by calendar period.

**Fig 3:**
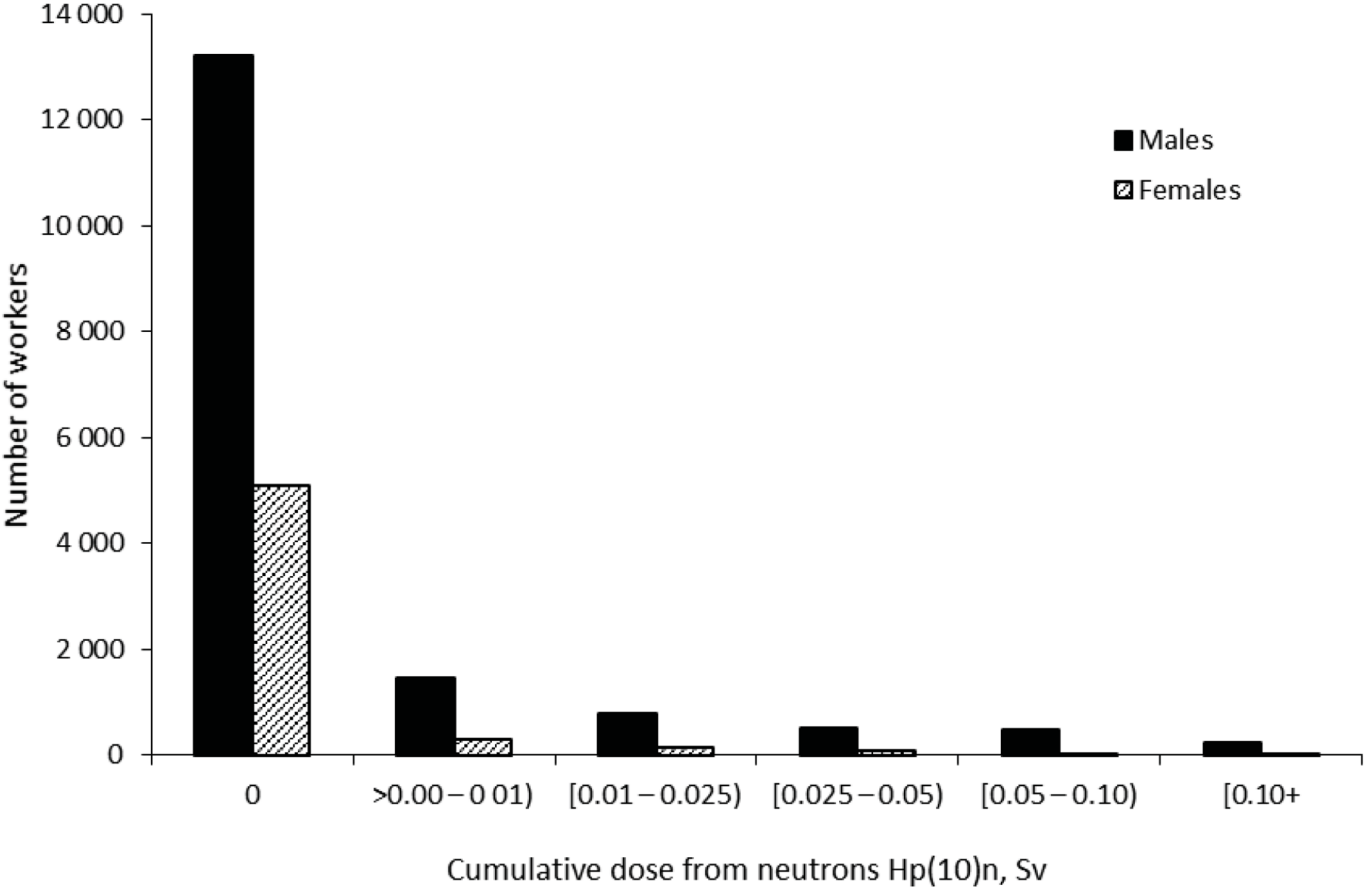
Distributions of workers from the study cohort depending on cumulative dose from neutrons.

For the study we used absorbed doses from external gamma-rays and neutrons provided by Mayak Worker Dosimetry System– 2008 (MWDS– 2008) developed within the framework of Russian-American collaboration [23]. MWDS– 2008 provides doses absorbed in 18 organs but, unfortunately, skin doses are not available so this study employed individual doses from homogeneous gamma-rays absorbed at a point of dosimeter fixation on a worker’s body at 10 mm depth [Hp(10) dose equivalent] (hereinafter ‘externa gamma-dose’) and individual doses from neutron exposure absorbed at a point of fixation of a radiation dosimeter on a body of a worker at 10 mm depth [*H*p(10)n dose equivalent] (hereinafter ‘neutron dose’) [24]. Cumulative dose from external γ-rays was 0.54 ± 0.76 Sv (95th percentile, 2.21 Sv; min–max: 0–8.43 Sv) in males and 0.44 ± 0.65 Sv (95th percentile, 1.87 Sv; min–max: 0–6.83 Sv) in females; mean annual doses (± SD) were 0.06 ± 0.13 Sv (95% percentile 0.28 Sv; min–max: 0–2.48 Sv) for males and 0.06 ± 0.11 Sv (95% percentile 0.27 Sv; min–max: 0–1.34 Sv) for females, correspondingly. The range of total doses was wide with 17% of the workers exposed to total external gamma-rays at levels higher than 1 Sv and 35% exposed to less than 0.1 Sv (Fig 1). The mean annual gamma-doses were the highest in the earliest years of the Mayak PA operation (1948–1953). The mean annual gamma-dose was 0.3 Sv/year in 1951, but decreased sharply over the next decade to 0.05 Sv/year by 1960. They continued to fall at a slower rate until 1980 after which the annual gamma-doses remained stable at around 0.008 Sv/year (Fig 2). Cumulative dose from neutrons was 0.034 ± 0.080 Sv (min–max: 0–2.64 Sv) in males and 0.033 ± 0.092 Sv (min–max: 0–1.15 Sv) in females (Fig 3).

### Statistical analysis

Data used in the present analysis were restricted to a period of residence in Ozyorsk because information on diseases, results of annual skin examinations and non-radiation factors was unavailable for migrants after they had left the city. Comparison was performed between groups within the study cohort. This study excluded 43 workers with acute radiation sickness due to high dose-rate gamma-neutron exposure, and also excluded 698 workers with missing medical information due to lost medical charts.

To run the analyses, the data were сompiled as multidimensional tables (S1 Table).

Utilizing conventional software Statistica 10.0, standardized incidence rates for MSN per 100 000 workers were calculated along with 95% confidence intervals (CI). Normalization was performed indirectly using an internal reference. A piecewise log-linear model called joinpoint analysis (Joinpoint Regression Program, version 4.0.4; Statistical Research and Applications Branch, National Cancer Institute), without constraints on the positions of the nodes or joinpoints was used to identify time trend changes and to estimate annual percent change (APC) in incidence rates. The model was specified to include joinpoints, which could occur in the middle of a period or between two consecutive periods. The model constrained the joinpoints to be at least one period and a half from each other, and at least two periods away from the start and end of the study. Under the assumption of heteroscedasticity and uncorrelated errors, the best-fitting model was searched on 4499 randomly permuted datasets using the grid search method. Tests, at an overall two-sided significance level of 0.10, were not adjusted for autocorrelation.

At the next stage of the analysis, relative risks (RR) were estimated for categories including one or a number of variables including adjustments for other variables. RR was computed based on maximum likelihood using AMFIT module of the EPICURE software [25]. 95% CIs for RR estimates and p values used to test statistical significance were computed using likelihood-based techniques integrated in the AMFIT module.

The first step was to investigate effects of various non-radiation factors on MSN incidence and the second step was to assess the effect of external gamma-ray exposure taking into account non-radiation factors and neutron exposure (via stratification). In addition to categorical analyses, incidence trends with radiation dose were tested using Poisson regression in AMFIT module of EPICURE software. In particular, excess relative risk per unit dose (ERR/Sv) was described with a linear trend with dose from external γ-rays including adjustment (via stratification) for non-radiation factors [sex, attained age (<20, 20–25,…, 80–85, >85 years) and calendar period (1946–1950, 1951–1955, 1956–1960, 1961–1965, …, 2011+)]. Namely, the used Poisson regression model was:

$$\lambda =\lambda_{0}\left(s,aa,cc\right)\cdot \left(1+\beta \cdot D\right)$$ (1)

where λ is incidence rate of MSN; λ_0_ is a background incidence of MSN; s is sex; aa is attained age; cc is calendar period; β is ERR/Sv; D is cumulative absorbed dose from external gamma-rays, Sv.

To investigate the effect of neutron exposure on the observed risk estimate a sensitivity analysis was performed. For the analysis dose from neutron exposure was considered as a categorical variable and was categorized as follows: <0.01, 0.01–0.025, 0.025–0.05, 0.05–0.10, >0.10, unmeasured 0.00. An adjustment for neutron dose was included via stratification. Thus, the Poisson regression models was as:

$$\lambda =\lambda_{0}\left(s,aa,cc,dn\right)\cdot \left(1+\beta \cdot D\right)$$ (2)

where dn is a cumulative dose from neutrons, Sv. Workers who were assumed to be unexposed to neutrons were not excluded from the analysis being categorized as ‘unmeasured 0.00’.

Such analysis design (including an adjustment for neutron dose via stratification) was chosen because doses from neutron exposure were measured only in 18.6% of the study cohort workers while omitting data on workers with unmeasured neutron doses would result in a considerable decrease of statistical power of the analysis.

Additionally, sensitivity analyses were performed to investigate effects of additional stratification by race, and of exclusion from the dataset workers employed at the facility less than one year, imposing various lag periods (0, 5, 10, 15 and 20 years) on doses from external gamma-rays. To lag doses from external γ-rays and neutrons, person-years from start of employment were taken into account while first *x* years from start of employment were included in zero category for gamma/neutron dose being lagged for *x* years.

Modification of radiation risk of MSN incidence due to sex, attained age and age at first employment at the Mayak PA and facility type was also investigated (while assessing heterogeneity and a log-linear trend of ERR/Sv with attained age). All significance tests were two-sided.

Data on facility type were taken into account over the entire follow-up period. ‘Plutonium production plant‘ category included workers who had ever been employed at the plutonium production plant, ‘radiochemical plant’ category included workers who had ever been employed at the radiochemical plant but had never been employed at the plutonium production plant and ‘reactor’ category included workers who had been employed at reactors but had never been employed at other facilities.

Data on smoking habits were taken into account over the entire follow-up period and estimated with a qualitative index. The qualitative index included values ‘unknown’, ‘never smoker’ and ‘ever smoker’. ‘Never smoker’ was assumed to be a worker who reported to have never smoked during a series of annual mandatory medical examinations.

Data on alcohol consumption were taken into account over the entire follow-up period and estimated with a qualitative parameter. ‘Non-drinker’ was assumed to be a worker who within the follow-up period during a series of annual mandatory medical examinations reported to have never drunk alcohol; ‘moderate drinker’ was assigned to a worker who choose this description to characterize his/her alcohol consumption habit during a series of annual mandatory medical examinations; ‘heavy drinker’ was assumed to be a worker in whose medical charts inebriety or chronic alcoholism were registered; ‘unknown’ was assigned to a worker with unavailable/missing information on this parameter.

## Results

**Fig 4:**
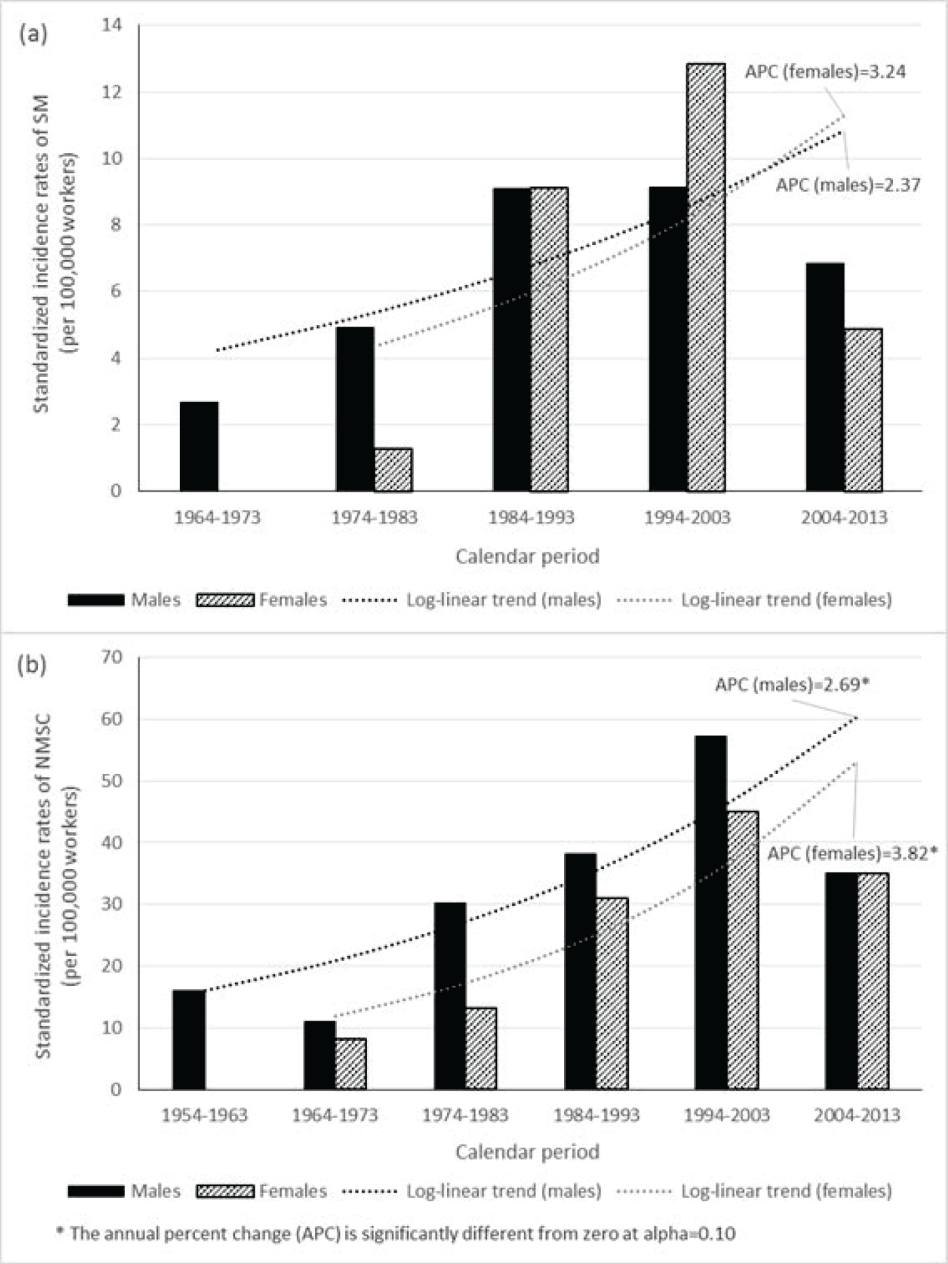
Standardized incidence rates of SM (a) and NMSC (b) in the study worker cohort.

Over the follow-up period in the study Mayak worker cohort 60 cases of SM and 294 cases of NMSC were registered within 571 462 and 565 019 person-years of follow-up, respectively. The vast majority of SM and NMSC cases were registered in workers at the age above 50 (85.0% and 86.4%, respectively). Age is known to be one of the main risk factors for malignant neoplasm development [3–5]. SM and NMSC cases were mostly registered during 1986–2013 period (86.7% and 80.3%, respectively), mainly, due to attained age of workers of the study cohort in this period. Standardized SM incidence rates were 8.51 ± 1.46 in males and 8.78 ± 2.27 in females per 100,000 workers while the corresponding rates for NMSC were 46.04 ± 3.40 and 37.40 ± 4.72, respectively (unpublished data in print). Standardized incidence rates for SM and NMSC in the study cohort markedly increased by the end of the follow-up (Fig 4). Significant log-linear trends were revealed for NMSC incidence rate increase by the end of the follow-up period both for males and females of the study cohort (APC = 2.69 and 3.82, respectively, P < 0.10) as well as the insignificant trend of SM incidence rate increase for male workers (APC = 2.37, P = 0.2) (Fig 4). The obtained results agree well with findings of other studies and support the common pattern of the increase of MSN incidence rate [1–3]. This upward trend for MSN incidence rate in Russia is driven by the increased expectation of life as well as by improvements in procedures needed to register the disease diagnosis.

Table 2 describes analysis of association of SM and NMSC incidence rates with non-radiation risk factors in the study cohort. NMSC incidence in females was significantly lower than in males (RR = 0.77; 95% CI: 0.60, 0.98). SM and MNSC incidence increased with increasing attained age both in males and females. For example, incidence RR in males older than 70 years for SM was 7.62 (95% CI: 2.30, 26.69), for NMSC was 17.70 (95% CI: 10.43, 30.67) as compared to the reference category including males aged <50 years. SM incidence risk in males first employed at one of the main production facilities of the Mayak PA at the age of 20–30 years was significantly decreased compared to those first employed at the age below 20 years. NMSC incidence risk was significantly lower in males first employed at the facility at the age above 30 years compared to those workers who had been first employed at the age below 20 years (Table 2). Calendar period of first employment at the facility was shown to have no significant effect on MSN incidence rates in the study worker cohort, but it was found that calendar period of the disease diagnosis had a significant effect in females. Specifically, SM incidence during 1976–1985 in females was significantly decreased when compared to the period of 1996–2005. Moreover, the analysis revealed that NMSC incidence was lower in reactor female workers than that in women employed at the radiochemical plant (RR = 0.54; 95% CI: 0.26, 0.99, p < 0.05). No significant association was shown for MSN incidence rates with the duration of employment at the Mayak PA. No influence of smoking and alcohol consumption on MSN incidence in the study cohort was shown while other studies report that MSN risk is increased in alcohol abusers who are at a higher risk of sunburn which, in its turn, is a factor inducing MSN development, what is suggested in numerous studies [23, 24]. MSN risk was increased in individuals with higher education degree and high social status with significant risk estimates obtained only for males diagnosed with NMSC (Table 2). For members of the study cohort this finding may be explained with more frequent and lengthy vacations spent in the southern regions where UVI index is times higher than in Ozyorsk. Additional risk analysis in relation to ethnicity demonstrated that NMSC risk in workers of the mixed race was lower than that in Caucasian race workers but the risk estimate was not significant likely due to the low number of cases in certain race groups (81% of cohort members are Caucasian).

**Table 2:** Skin cancer: Non-radiation factor analysis.

Factors		SM		NMSC	
		RR (95% CI)	Number of cases	RR (95% CI)	Number of cases
*Sex*					
Males		1	37	1	187
Females		1.03 (0.59, 1.76)	23	**0.77 (0.60, 0.98)**	107
*Attained age (wider categories compared to those used for stratification)*					
Males	<50	1	6	1	30
	[50–60)	**4.40 (1.66, 13.08)**	13	**4.49 (2.76, 7.46)**	48
	[60–70)	**4.92 (1.67, 15.78)**	10	**8.35 (5.07, 14.05)**	56
	[70+	**7.62 (2.30, 26.69)**	8	**17.70 (10.43, 30.67)**	53
Females	<50	1	3	1	10
	[50–60)	0.68 (0.12, 3.70)	3	2.07 (0.86, 5.20)	14
	[60–70)	1.41 (0.38, 6.84)	8	**3.42 (1.43, 8.84)**	24
	[70+	1.59 (0.38, 8.43)	9	**9.59 (4.04, 25.10)**	59
*Age at first employment*					
Males	<20	1	16	1	50
	[20–30)	**0.42 (0.18, 0.96)**	13	0.82 (0.56, 1.20)	101
	[30+	0.61 (0.18, 1.77)	8	**0.53 (0.30, 0.92)**	36
Females	<20	1	3	1	20
	[20–30)	1.63 (0.51, 7.30)	16	0.68 (0.40, 1.21)	48
	[30+	0.45 (0.08, 2.58)	4	0.81 (0.45, 1.47)	39
*Period of first employment*					
Males	1948–1958	1	20	1	114
	1959–1982	0.64 (0.26, 1.49)	17	0.86 (0.56, 1.15)	73
Females	1948–1958	1	13	1	67
	1959–1982	0.42 (0.11, 1.35)	10	0.90 (0.56, 1.42)	40
*Calendar period of the disease diagnosis (wider categories compared to those used for stratification)*					
Males	1946–1955	-	0	1.20 (0.19, 4.08)	2
	1956–1965	-	0	0.33 (0.05, 1.12)	2
	1966–1975	0.25 (0.01, 1.37)	1	0.84 (0.42, 1.58)	12
	1976–1985	0.75 (0.25, 2.04)	6	0.94 (0.58, 1.50)	28
	1986–1995	0.96 (0.41, 2.22)	11	0.93 (0.62, 1.37)	44
	1996–2005	1	12	1	59
	2006–2013	0.80 (0.29, 2.04)	7	0.90 (0.59, 1.35)	40
Females	1946–1955	-	0	-	0
	1956–1965	-	0	-	0
	1966–1975	-	0	0.87 (0.29, 2.37)	7
	1976–1985	**0.14 (0.01, 0.92)**	1	0.45 (0.17, 1.05)	7
	1986–1995	0.80 (0.27, 2.33)	7	0.74 (0.41, 1.29)	20
	1996–2005	1	9	1	44
	2006–2013	1.16 (0.37, 3.36)	6	0.89 (0.54, 1.45)	29
*Facility type*					
Males	Reactors	1.43 (0.62, 3.22)	11	1.02 (0.71, 1.45)	49
	Radiochemical plant	1	13	1	79
	Plutonium production plant	1.24 (0.57, 2.72)	13	1.02 (0.72, 1.43)	59
Females	Reactors	0.71 (0.20, 2.06)	4	**0.54 (0.26, 0.99)**	11
	Radiochemical plant	1	12	1	50
	Plutonium production plant	0.62 (0.23, 1.56)	7	1.19 (0.79, 1.79)	46
*Duration of employment*					
Males	< 1	-	0	1.29 (0.49, 2.82)	6
	[1–10)	1	4	1	39
	[10+	1.92 (0.75, 6.48)	33	0.93 (0.65, 1.37)	142
Females	< 1	-	0	1.04 (0.25, 2.94)	3
	[1–10)	1	9	1	29
	[10+	0.48 (0.20, 1.21)	14	1.14 (0.74, 1.81)	75
*Alcohol consumption*					
Males	Non-drinker	1	2	1	4
	Moderate drinker	0.44 (0.13, 2.72)	25	1.12 (0.47, 3.65)	126
	Heavy drinker	0.34 (0.09, 2.20)	10	0.87 (0.36, 2.90)	50
	Unknown	-	0	1.04 (n/a, n/a)	7
Females	Non-drinker	1	13	1	58
	Moderate drinker	0.50 (0.18, 1.26)	7	1.07 (0.71, 1.58)	47
	Heavy drinker	-	0	0.37 (0.02, 1.67)	1
	Unknown	3.00 (0.67, 9.60)	3	0.27 (0.02, 1.24)	1
*Smoking*					
Males	Never smoker	1	14	1	45
	Ever smoker	0.50 (0.26, 1.00)	23	0.92 (0.66, 1.31)	142
	Unknown	-	0	-	0
Females	Never smoker	1	21	1	102
	Ever smoker	0.80 (0.04, 4.04)	1	0.88 (0.27, 2.13)	4
	Unknown	2.67 (0.145, 13.51)	1	0.56 (0.03, 2.54)	1
*Education*					
Males	Non-higher education	1	22	1	108
	Higher education	1.71 (0.85, 3.33)	14	**1.51 (1.10, 2.06)**	63
	Unknown	0.44 (0.02, 2.20)	1	1.02 (0.57, 1.72)	16
Females	Non-higher education	1	16	1	84
	Higher education	2.78 (0.96, 7.04)	6	1.29 (0.67, 2.29)	12
	Unknown	0.99 (0.05, 5.19)	1	1.65 (0.81, 3.05)	11
*Race*					
Males	Caucasian	1	34	1	170
	Mixed	-	0	0.99 (0.39, 2.06)	6
	Mongoloid	-	0	-	0
	Unknown	0.75 (0.18, 2.10)	3	0.59 (0.30, 1.04)	11
Females	Caucasian	1	20	1	97
	Mixed	-	0	0.29 (0.02, 1.30)	1
	Mongoloid	-	0	-	0
	Unknown	1.27 (0.30, 3.73)	3	0.80 (0.38, 1.50)	9

Table 3 summarizes results of categorical analyses of MSN incidence in the study worker cohort. SM incidence risk was increased in all external gamma-dose categories compared to the reference category (0–0.05 Sv cumulative doses), but no significance level was achieved for RR estimates what could be explained by the small number of cases and the low statistical power of the analysis. Meanwhile, a significantly increased risk was found for NMSC incidence in workers occupationally exposed to radiation at cumulative dose above 2.0 Sv (RR = 2.52; 95% CI: 1.60, 3.97) when compared to the reference category (0–0.05 Sv cumulative dose).

**Table 3:** RR of skin cancer incidence by cumulative dose from external gamma-ray exposure.

Cumulative dose from external gamma-rays (Sv), range	Mean cumulative dose from external gamma-rays (Sv)	Person-years of the follow-up	Number of skin cancer cases	RR (95% CI)
Melanoma (SM)				
[0–0.05)	0.019	128 341	9	1
[0.05–0.10)	0.074	67 688.6	9	1.50 (0.59, 3.86)
[0.10–0.50)	0.232	189 455	16	1.01 (0.43, 2.43)
[0.50–1.00)	0.697	73 331.4	12	2.26 (0.88, 6.04)
[1.00–2.00)	1.365	63 629.7	11	2.43 (0.91, 6.75)
≥2.00	2.582	30 776	3	1.27 (0.26, 4.81)
Non-melanoma skin cancer (NMSC)				
[0–0.05)	0.019	127 188	42	1
[0.05–0.10)	0.074	67 356	30	1.16 (0.72, 1.85)
[0.10–0.50)	0.233	18 7887	79	0.84 (0.57, 1.24)
[0.50–1.00)	0.699	72 262.6	49	1.17 (0.76, 1.81)
[1.00–2.00)	1.370	62 600.3	45	1.21 (0.78, 1.89)
≥2.00	2.582	29 648.2	44	**2.52 (1.60, 3.97)**

**Fig 5:**
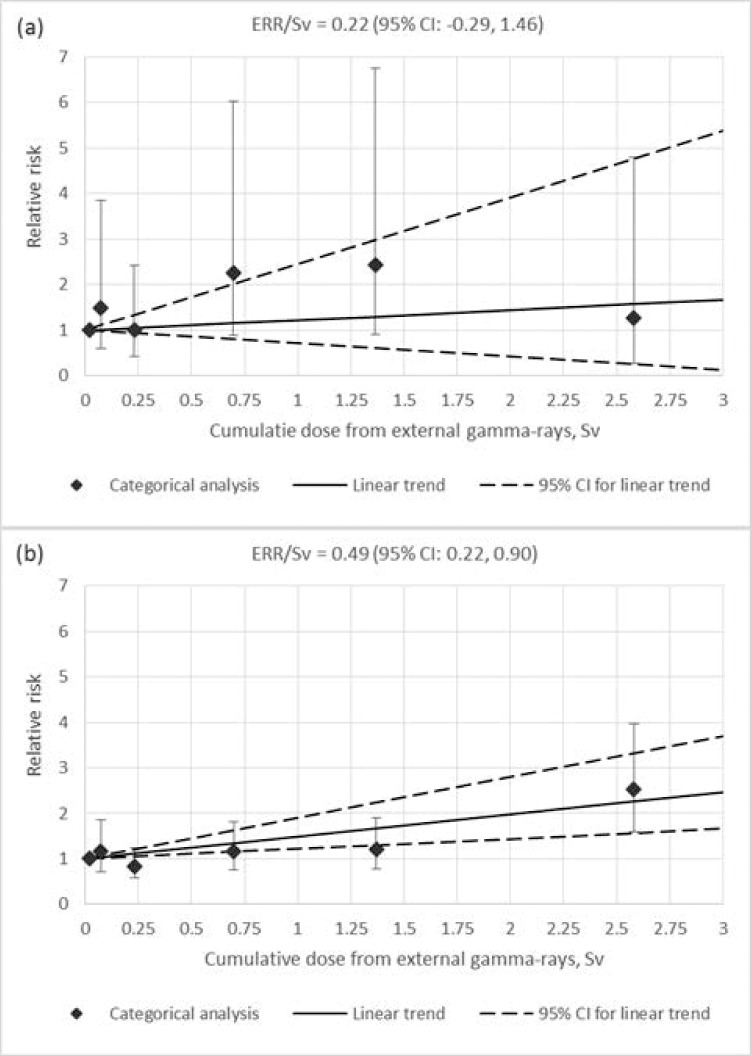
Relative risk of SM (a) and NMSC (b) incidence by cumulative dose from external gamma-rays.

Table 4 and Fig 5 demonstrate an excess relative risk per unit dose (ERR/1.0 Sv) for MSN incidence associated with dose from external gamma-rays based on the linear risk model. NMSC incidence was found to be significantly associated with cumulative dose from external gamma-rays with ERR/Sv of 0.49 (95% CI: 0.22, 0.90) unadjusted for neutron dose and neutron dose adjusted ERR/Sv of 0.51 (95% CI: 0.22, 0.93). The risk estimate increased with increasing lag period and with 20 year lag period imposed it was 0.60 (95% CI: 0.28, 1.05). Significant ERR/Sv was estimated for NMSC incidence associated with external gamma rays exposure in male workers of the study cohort (0.70; 95% CI: 0.28, 1.41), but not in female workers, however, no significant differences were revealed between them (p = 0.164). ERRs/Sv of external exposure for NMSC incidence were significant in all age categories excluding the group of workers at the age above 70 years, and differences among age categories were significant (p = 0.024). NMSC incidence risk decreased with increasing attained age but this trend was insignificant (p = 0.252). The risk was found to be modified neither by age at first employment at the Mayak PA (p > 0.5), nor by facility type (p > 0.5).

**Table 4:** Excess relative risk of skin cancer incidence by cumulative dose from external gamma-rays exposure.

Analysis type	ERR/Sv (95% confidence interval)	
	SM	NMSC
Main analysis, 0 year lag	0.22 (-0.29, 1.46)	0.49 (0.22, 0.90)
Main analysis, 5 year lag	0.22 (-0.29, 1.44)	0.49 (0.21, 0.89)
Main analysis, 10 year lag	0.22 (-0.29, 1.42)	0.50 (0.22, 0.91)
Main analysis, 15 year lag	0.24 (-0.29, 1.47)	0.52 (0.23, 0.93)
Main analysis, 20 year lag	0.32 (-0.28, 1.76)	0.60 (0.28, 1.05)
Sensitivity analysis–additional stratification by neutron dose	0.15 (-0.41, 1.31)	0.51 (0.22, 0.93)
Sensitivity analysis–additional stratification by race	0.17 (-0.40, 1,38)	0.48 (0.20, 0.88)
Sensitivity analysis excluding workers employed less than one year	0.16 (-0.39, 1.29)	0.51 (0.23, 0.94)
*Analysis restricted to include only*:		
Male workers	-0.06 (n/a, 0.82)	0.70 (0.28, 1.41)
Female workers	2.18 (n/a, 15.22)	0.22 (-0.09, 0.77)
Test for heterogeneity between males and females	p_1_ = 0.075	p_1_ = 0.164
*Attained age*		
< 50 years	-0.09 (n/a, 4.81)	0.99 (0.06, 3.45)
50–59 years	-0.17 (n/a, 0.61)	2.17 (0.51, 8.63)
60–69 years	1.09 (-0.46, 8.27)	1.32 (0.45, 3.36)
70 + years	1.04 (-0.86, 41.84)	0.16 (-0.10, 0.60)
Test for heterogeneity among groups of workers of different attained age	p_2_ = 0.460	p_2_ = 0.024
Test for log-linear trend in ERR/Sv by attained age	p_3_ > 0.5	p_3_ = 0.252
*Age at first employment*		
<20 years	2.41 (-0.94, 24.85)	0.61 (0.03, 2.12)
20–29 years	0.21 (-0.51, 2.36)	0.39 (0.06, 0.98)
30+ years	-0.18 (n/a, 0.62)	0.91 (0.09, 2.95)
Test for heterogeneity among groups of workers by age at first employment	p_4_ = 0.396	p_4_ > 0.5
*Facility type*		
Reactors	0.26 (-0.56, 4.51)	0.35 (-0.21, 1.78)
Radiochemical plant	0.38 (-0.68, 5.80)	0.93 (0.32, 2.29)
Plutonium production plant	-0.01 (-1.77, 6.27)	0.72 (0.10, 1.86)
Test for heterogeneity among groups of workers by facility types	p_5_ > 0.5	p_5_ > 0.5

Note: n/a refers to non-identifiable bounds of confidence intervals

The analysis results did not demonstrate a significant association of SM incidence with cumulative dose from external gamma-rays; ERR/Sv unadjusted for neutron dose was 0.22 (95% CI: -0.29, 1.46) and 0.15 (95% CI: -0.41, 1.31) with an adjustment for neutron dose included (Table 4). It should be noted that the risk increased with increasing lag periods but remained insignificant (0.32 95% CI: –0.28, 1.76 with 20-year lad period imposed). All risk estimates fell within wide confidence intervals due to a small number of SM cases identified in the study worker cohort. Exclusion of individuals who were working at the Mayak PA less than 1 year from the analyzed dataset did not affect the results obtained for the whole cohort.

## Discussion

Results of the current study provide evidence to association of MSN incidence in the cohort of Mayak PA workers with sex, attained age, age at first employment at the facility, facility type and education as well as to the fact that MSN incidence was not dependent on calendar period of employment, calendar period of diagnosis, duration of employment, alcohol consumption and smoking. The increase of MSN incidence rates with increasing attained age could be expected and is driven by the age causation of the disease. Information on role of other non-radiation factors (for example, smoking, alcohol consumption) for MSN development reported in different studies is inconsistent [26–28]. Meanwhile results of epidemiological and clinical studies prove that UVI is the main ambient factor, which increases MSN risk [3, 6, 7]. Within the present retrospective study, we could not investigate sufficiently such factor as UVI. However, the risk analysis conducted for various MSN sites based on the study cohort data showed that incidence rates for SM and NMSC localized in face and neck regions were significantly decreased compared to body localizations while other studies demonstrated that if the UVI index was high, then risk of facial and neck MSN increased significantly [29]. It should be noted that all workers of the study cohort were living in the city of Ozyorsk in the Southern Urals in the same climate with the low index of UVI over the whole follow-up period. Also, it is worth noting that all workers of the study cohort were working only indoors.

It is well known that pre-malignant skin lesions and actinic keratoses increase skin cancer risks [30, 31]. However, since these cutaneous changes were very rare in members of the study cohort, they were not included in the analysis because of the insufficient statistical power.

As a result of the study we found a significantly increased risk of NMSC (but not SM) incidence in workers occupationally exposed to radiation at doses above 2.0 Sv accumulated over prolonged periods.

Dose-response analysis taking into account non-radiation factors (sex, attained age and calendar period) and neutron doses did not reveal a significant association of SM incidence with cumulative dose form external gamma-rays, likely due to a small number of cases and, hence, the low statistical power of the study. However, NMSC incidence was found to be significantly associated with cumulative dose from external gamma-rays both unadjusted and adjusted for neutron dose. Moreover, a sensitivity analysis demonstrated that ERR/Sv of external radiation for NMSC incidence increased after the adjustment for neutron dose was included. The risk estimated increased with the increasing lag period. ERR/Sv was found to be significantly modified neither by sex, nor by age at first employment at the facility, nor by facility type. However, the risk was shown to be significantly modified by attained age. ERR/Sv of external radiation for NMSC incidence in the group of workers aged <50 years was 0.99 (95% CI: 0.06, 3.45), in 50–59 years age group it was 2.17 (95% CI: 0.51, 8.63), in 60–69 years age group it was 1.32 (95% CI: 0.45, 3.36) and in age group of >70 years it was 0.16 (95% CI: -0.10, 0.60).

NMSC risks estimated in the present study (0.49; 95% CI: 0.22, 0.90) were lower than the corresponding risks estimated for atomic bomb survivors (0.72; 95% CI: 0.36, 1.2) [9]. These differences were mainly driven by strongly varying exposure scenarios (acute radiation exposure due to atomic bombings to the skin cover were higher than the corresponding doses accumulated by the study cohort members). Meanwhile, the non-melanoma incidence risk obtained in the present study was notably higher than that in members of US radiologist cohort, specifically, for basal cell carcinomas (ERR/Gy = 0.03, 95% CI: -0.39, 0.56) [11].

Similarly to the present study, none of main epidemiological studies demonstrated a significant association of SM with radiation dose, except for US radiologist cohort study analyzing effects of exposure following fluoroscopy interventional procedures and revealing a borderline significant increased risk of SM incidence (HR = 1.16, 95% CI: 1.02, 1.32) [13]. However, the authors concluded that this reported result could be of chance given a small number of SMs in this cohort.

The large size of the study cohort, long follow-up period (approximately 70 years), and available information on individually measured external gamma doses and sufficient statistical power of the study may be regarded as its strengths. One of the main advantages of the study cohort is the fact that dermatologist checkups and skin examinations were mandatory during annual medical examinations of the study cohort workers. Meanwhile we acknowledge that screening effect is typical for incidence studies of the Mayak PA workers. This effect is evidenced by the observation of higher SM and NMSC incidence rates among the cohort members than the corresponding rates among population of the Russian Federation and of the Urals Federal District (the region where Ozyorsk city and the Mayak PA are located) [32,33]. Meanwhile it should be highlighted that all workers, regardless of sex, age, working site, occupation, radiation type and dose, etc., were mandatorily subjected to medical health examinations following the standard unified protocol what excludes the possibility for self-selection (e.g. due to ill health) and dose-selection biases. Additionally, it should be emphasized that medical doctors who performed these mandatory health examinations had no access to information on radiation doses of workers.

The main limitation of the study is unavailable estimates of skin doses in the Mayak worker dosimetry system MWDS-2008 [23], but in future detailed occupation exposure routes, individual doses from external gamma-rays measured with personal film badges, detailed radiation exposure scenarios as well as occupation data will enable skin dose reconstruction and reanalysis of risk for the Mayak worker cohort based on an extended follow-up period and taking into account histology types of malignant skin neoplasms.

## Conclusion

The study results demonstrated that SM and NMSC incidence was dependent on sex, attained age, age at first employment at the enterprise, type of facility, education level and was not dependent on calendar period of first employment, calendar period of diagnosis, duration of employment, smoking and alcohol consumption statuses. The risk of NMSC incidence was found to be significantly increased in workers occupationally exposed to ionizing radiation at cumulative doses above 2.0 Sv (RR = 2.52; 95% CI: 1.60, 3.97) compared to a reference dose category (0–0.05 Sv). NMSC incidence was found to be significantly associated with cumulative external gamma-dose with ERR/Sv of 0.49 (95% CI: 0.22, 0.90) without an adjustment for neutron dose and 0.51 (95% CI: 0.22, 0.93) while adjusted for neutron dose. Results of the analysis did not reveal a significant association of SM incidence with cumulative dose from external gamma-rays with ERR/Sv of 0.22 (95% CI: -0.29, 1.46) not including a neutron dose adjustment and of 0.15 (95% CI: -0.41, 1.31) while adjusted for dose from neutron exposure.

## Supporting information

S1 Table Variables used in the analyses. Azizova_Skin Ca_Suppl.
(DOCX)
