# Supplementary material for: Risk of malignant skin neoplasms in a cohort of workers occupationally exposed to ionizing radiation at low dose rates
Source: PLoS One. 2018 Oct 5;13(10):e0205060. doi: 10.1371/journal.pone.0205060 (PMC6173419; doi:10.1371/journal.pone.0205060)
Supplement: S1 Table — Azizova_Skin Ca_Suppl. (DOCX) [file pone.0205060.s001.docx]

**S1 Table. Variables used in the analyses**

| Variable type | Variable name | Description | |
| --- | --- | --- | --- |
| Fixed | Sex | 1: Males | 2: Females |
| Fixed | Period of first employment | 1: 1948–1958  2: 1958–1982 |  |
| Fixed | Age at first employment | 1: <20  2: 20–29  3: 30+ |  |
| Fixed | Smoking status | 1: Never smoker  2: Ever smoker | 3: Unknown |
| Fixed | Alcohol consumption status | 1: Never drinker  2: Moderate drinker | 3: Heavy drinker  4: Unknown |
| Fixed | Education | 1: Non-higher education  2: Higher education | 3: Unknown |
| Fixed | Facility type | 1: Reactors  2: Radiochemical plant | 3: Plutonium production plant |
| Time-dependent | Attained age | 1: 15–20  2: 20–25  3: 25–30  4: 30–35  5: 35–40  6: 40–45  7: 45–50  8: 50–55 | 9: 55–60  10: 60–65  11: 65–70  12: 70–75  13: 75–80  14: 80–85  15: 85+ |
| Time-dependent | Calendar period | 1: 1946–1950  2: 1951–1955  3: 1956–1960  4: 1961–1965  5: 1966–1970  6: 1971–1975  7: 1976–1980 | 8: 1981–1985  9: 1986-1990  10: 1991–1995  11: 1996–2000  12: 2001–2005  13: 2006–2010  14: 2011–2013 |
| Time-dependent | Duration of employment | 1: <1  2: 1–10 | 3: >10 |
| Time-dependent | Cumulative external gamma-dose (Sv) | 1: 0–0.05  2: 0.05–0.10  3: 0.10–0.50 | 4: 0.50–1.00  5: 1.00–2.00  6: ≥2.00 |
| Time-dependent | Cumulative neutron dose (Sv) | 1: 0.00  2: >0.00–0.01  3: 0.01–0.025 | 4: 0.025–0.05  5: 0.05–0.10  6: ≥0.10 |
| Estimated | Attained age | Person-years weighted by attained age | |
|  | Cumulative external gamma-dose (Sv) | Person-years weighted by cumulative external gamma-dose | |
|  | Person-years* | Person-years at risk | |
| Number of cases | Skin cancer type* | SM | |
|  |  | NMSC | |

Note: * in relation to skin cancer type.
